# Nonalcoholic Wernicke encephalopathy with petechial hemorrhage in the tectal region

**DOI:** 10.1055/s-0043-1770347

**Published:** 2023-08-30

**Authors:** Gabriel de Deus Vieira, Augusto Celso Scarparo Amato Filho, Alfredo Damasceno

**Affiliations:** 1 Universidade de Campinas, Departamento de Neurologia, Campinas SP, Brazil.; 2 Universidade de Campinas, Departamento de Neuroradiologia, Campinas SP, Brazil.

A 39-year-old woman, after an episode of acute appendicitis, developed necrosis and intestinal obstruction, requiring colectomy and ileostomy. After hospital discharge, she had a high output through the ileostomy and low food intake. A few months later, she developed bilateral nystagmus, ataxic syndrome and difficulty concentrating. Susceptibility weighted imaging (SWI) sequence of the brain magnetic resonance imaging (MRI) showed hypointensities in the mammillary bodies and inferior colliculi, which might represent microbleeds ( Figure 1 ).

**Figure 1:**
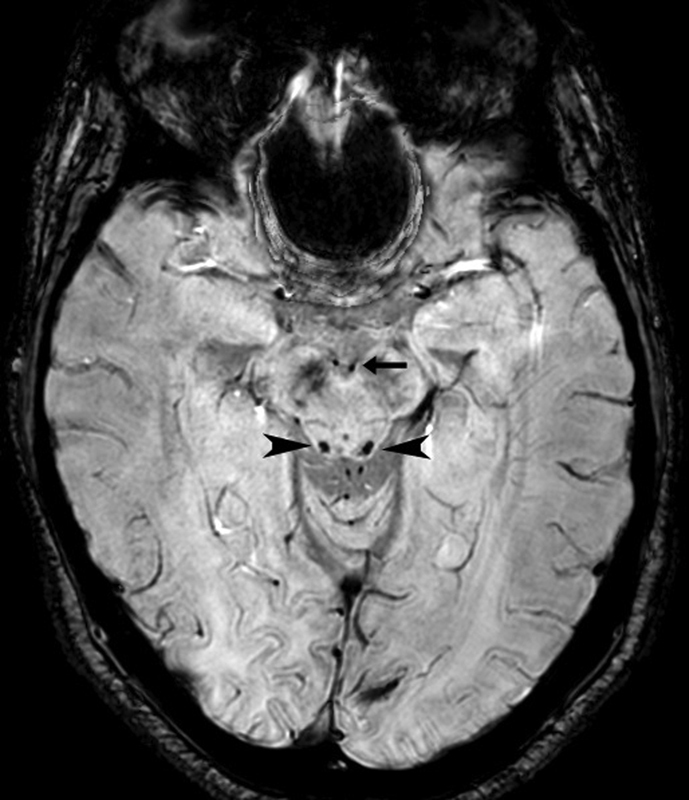
SWI sequence: focal hypointensities in the mammillary bodies (straight arrow) and mesencephalic quadrigeminal plate, notably in the inferior colliculi (sharp arrows).

Wernicke encephalopathy (WE) usually affects the mamillary bodies, thalami, and periaqueductal region. Rare hemorrhagic manifestations are reported in the literature, most in the mamillary bodies. The breakdown of the blood-brain barrier may contribute to this petechial hemorrhage in WE. 1 2

## References

[1] Zuccoli G Santa Cruz D Bertolini M MR imaging findings in 56 patients with Wernicke encephalopathy: nonalcoholics may differ from alcoholics AJNR Am J Neuroradiol 2009 30 01 171 176 18945789 10.3174/ajnr.A1280 PMC7051709

[2] Hattingen E Beyle A Müller A Klockgether T Kornblum C Wernicke encephalopathy: SWI detects petechial hemorrhages in mammillary bodies in vivo Neurology 2016 87 18 1956 1957 27799475 10.1212/WNL.0000000000003294

